# Supplementary figures and images for: Transcriptome Analysis Revealed a Positive Role of Ethephon on Chlorophyll Metabolism of Zoysia japonica under Cold Stress
Source: Plants (Basel). 2022 Feb 5;11(3):442. doi: 10.3390/plants11030442 (PMC8839986; doi:10.3390/plants11030442)

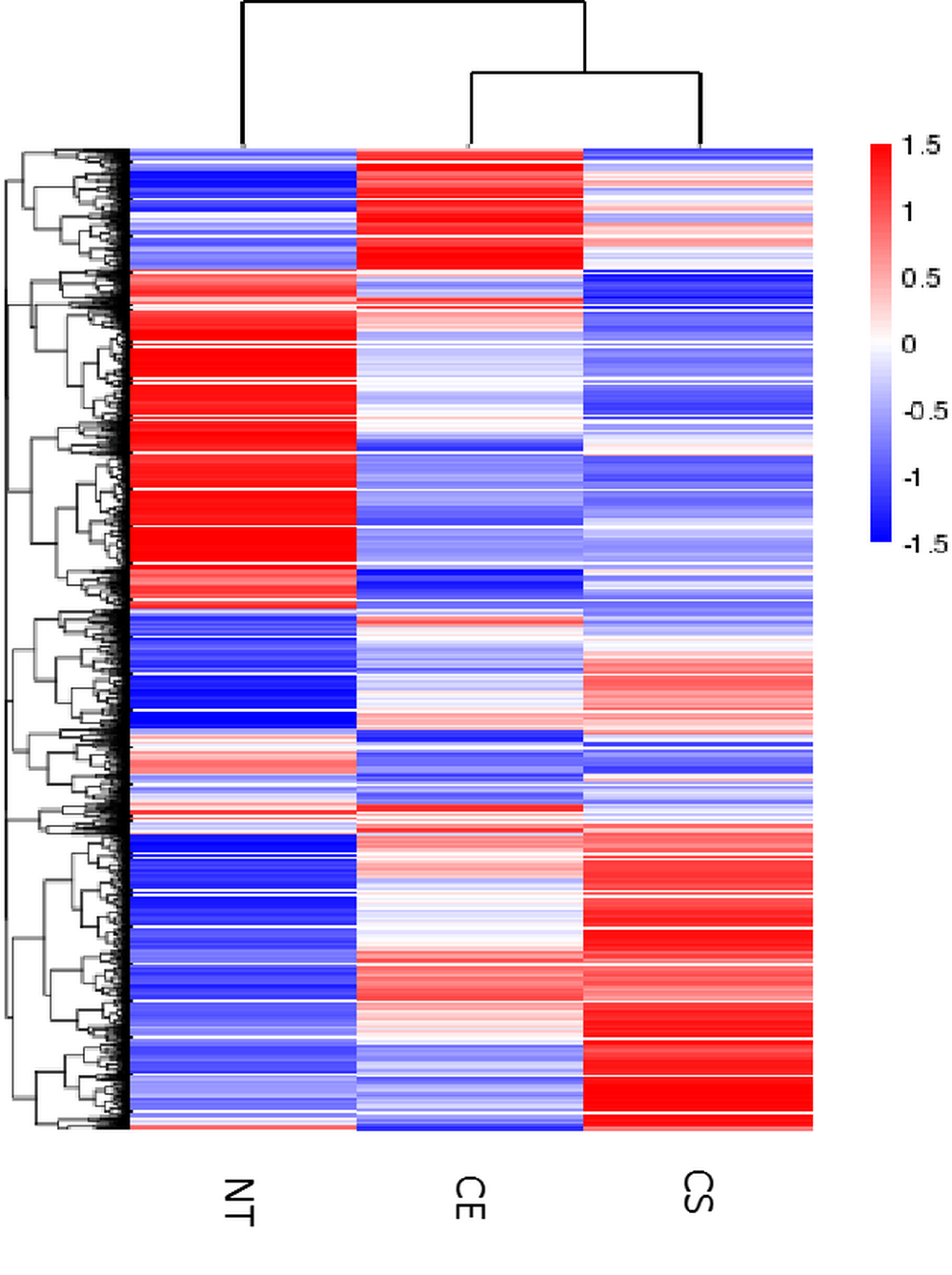

Supplement: Supplementary file 1 [file plants-11-00442-s001.zip › Figure S1. Heat map of all differentially expressed genes in Z. japonica.tif]
